# Identification of lncRNAs Deregulated in Epithelial Ovarian Cancer Based on a Gene Expression Profiling Meta-Analysis

**DOI:** 10.3390/ijms241310798

**Published:** 2023-06-28

**Authors:** Martín Salamini-Montemurri, Mónica Lamas-Maceiras, Lidia Lorenzo-Catoira, Ángel Vizoso-Vázquez, Aida Barreiro-Alonso, Esther Rodríguez-Belmonte, María Quindós-Varela, M. Esperanza Cerdán

**Affiliations:** 1 Centro Interdisciplinar de Química e Bioloxía (CICA), As Carballeiras, s/n, Campus de Elviña, Universidade da Coruña, 15071 A Coruña, Spain; monica.lamas@udc.es (M.L.-M.); lidia.lorenzo.catoira@udc.es (L.L.-C.); a.vizoso@udc.es (Á.V.-V.); aida.barreiro@udc.es (A.B.-A.); esther.belmonte@udc.es (E.R.-B.); 2 Facultade de Ciencias, A Fraga, s/n, Campus de A Zapateira, Universidade da Coruña, 15071 A Coruña, Spain; 3 Instituto de Investigación Biomédica de A Coruña (INIBIC), As Xubias de Arriba 84, 15006 A Coruña, Spain; maria.quindos.varela@sergas.es; 4 Complexo Hospitalario Universitario de A Coruña (CHUAC), Servizo Galego de Saúde (SERGAS), 15006 A Coruña, Spain

**Keywords:** long non-coding RNAs (lncRNAs), epithelial ovarian cancer (EOC), transcriptomics, meta-analysis

## Abstract

Epithelial ovarian cancer (EOC) is one of the deadliest gynecological cancers worldwide, mainly because of its initially asymptomatic nature and consequently late diagnosis. Long non-coding RNAs (lncRNA) are non-coding transcripts of more than 200 nucleotides, whose deregulation is involved in pathologies such as EOC, and are therefore envisaged as future biomarkers. We present a meta-analysis of available gene expression profiling (microarray and RNA sequencing) studies from EOC patients to identify lncRNA genes with diagnostic and prognostic value. In this meta-analysis, we include 46 independent cohorts, along with available expression profiling data from EOC cell lines. Differential expression analyses were conducted to identify those lncRNAs that are deregulated in (i) EOC versus healthy ovary tissue, (ii) unfavorable versus more favorable prognosis, (iii) metastatic versus primary tumors, (iv) chemoresistant versus chemosensitive EOC, and (v) correlation to specific histological subtypes of EOC. From the results of this meta-analysis, we established a panel of lncRNAs that are highly correlated with EOC. The panel includes several lncRNAs that are already known and even functionally characterized in EOC, but also lncRNAs that have not been previously correlated with this cancer, and which are discussed in relation to their putative role in EOC and their potential use as clinically relevant tools.

## 1. Introduction

Epithelial ovarian cancer (EOC) is the second most common cause of death due to gynecological cancers, with approximately 314,000 new cases and 205,000 deaths worldwide in 2020, and with increasing trends predicted [1]. EOC patients are usually diagnosed at an advanced stage of the disease due to the initially asymptomatic character of the tumor, leading to a dramatic five-year overall survival rate below 40% [2]. An early diagnosis correlates with a better prognosis but, unfortunately, an efficient, approved, and easy protocol based on biomarkers is not available for EOC [3]. Long non-coding RNAs (lncRNAs) are transcripts longer than 200 nucleotides that regulate gene expression at different levels, taking part in physiological and pathological processes, including EOC [4]. More than 150 lncRNAs have been studied and related to EOC so far [5,6,7]. Transcriptome-wide approaches, such as expression microarrays and RNA deep sequencing, produce a large amount of information that is shared with the scientific community thanks to online repositories, such as the Gene Expression Omnibus (GEO) or the European Nucleotide Archive [8]. There are many gene expression profiling studies in the context of EOC, which also provide clinical information about patients and, depending on the cohort size, are able to capture the patient-to-patient variability, thereby allowing the validation and discovery of biological markers and the advance of precision medicine [9]. Several transcriptomic meta-analyses have been previously published analyzing these data in EOC; however, they either used a small number of studies, those available at the moment [10,11,12,13], or were focused on protein-coding genes and neglected lncRNAs [14,15,16,17]. The present work is the first lncRNA meta-analysis in EOC comprising a high and significant number of studies—specifically, 46 independent cohorts—and the first to integrate information from expression microarrays and bulk RNA sequencing. The objective of this work is to reanalyze and compare all of the EOC-patient-derived microarray and RNA sequencing studies available to date, in order to find lncRNAs that highly correlate to clinical aspects and that might have clinical application in the management of EOC patients.

## 2. Results

In this meta-analysis, we analyzed publicly available independent transcriptomic datasets from EOC-patient-derived samples, which contain lncRNA expression data as well as associated clinical data from patients included in the studies. They are representative of the actual clinical knowledge on EOC and integrate the wide variability between individuals and cohorts. 

In our search for gene expression profiling data in ovarian cancer, we initially found a total of 63 studies, of which 51 and 12 accounted for microarray and bulk RNA-Seq technologies, respectively. In terms of the type of analyzed sample, 48 studies used tissue, 2 blood, 2 blood serum, 1 urine, and 1 saliva, whereas 9 worked with cell lines (2 of them including their derived exosomes). All of the studies were considered epithelial ovarian cancer cases, although four of them contained additional data from stromal cells from the tumor that were not used in this meta-analysis. Seventeen studies restrained the cancer samples to one specific subtype, such as high-grade serous or clear cell; another six compared several subtypes within the same study, while the remaining thirty-eight studies did not specify the EOC subtype. Those samples named in the original studies as “serous”, “high-grade serous”, or “low-grade serous” were jointly considered. Out of the 51 studies with cancer patients (excluding cell line studies) who were naïve to treatment at the time of sample extraction, 22 of them did not include samples from healthy tissue for comparison, but 7 of these 22 studies included either cancer cells from ascites and/or peritoneal metastatic samples matched to the primary tumor. There were 11 studies with prognostic information, including survival and/or progression over time. Twelve studies only considered miRNAs, due to the selected microarray platform. The full list of initially identified studies, including the cohort size of each study, can be found in the Supplementary Materials (Table S1).

Our objective was to identify lncRNAs (antisense, sense, intronic, intergenic, divergent, overlapping, and non-overlapping) with clinical value related to epithelial ovarian cancer. Thus, we discarded 17 studies from the 63 initially identified according to the criteria specified in Section 4, thereby excluding studies with information only from miRNAs and/or carried out only with cell lines. Due to the heterogeneity of clinical information available in each study, we performed different rounds of analyses focusing on different studies, which included information of relevance in diagnosis or prognosis, correlation to metastasis, chemotherapy resistance, and histological EOC subtypes. Therefore, in the following sections, we use the same criteria to present the results of the different analyses performed.

### 2.1. Analysis of lncRNAs with Putative Diagnostic Value in EOC

In this first round of our analysis, we sought to identify differentially expressed lncRNAs—either upregulated or downregulated—in EOC compared with healthy samples. For this category, we considered 24 studies that used both cancer and control samples; however, EGAD00001000877 data are not publicly available, and the RNA-Seq libraries from GSE192410 were prepared from circular RNA only; therefore, these data were discarded. We analyzed the remaining 22 studies, whose metadata and results for the differential expression analyses are available in the Supplementary Materials (Table S2). The RNA analyzed in these studies was derived from tissue samples, except for GSE29220, whose RNA was derived from saliva samples. Only one study, GSE137238, presented matched cancer and normal ovarian tissue from the same woman, whereas in the rest, the case and control samples came from different women.

**Figure 1:**
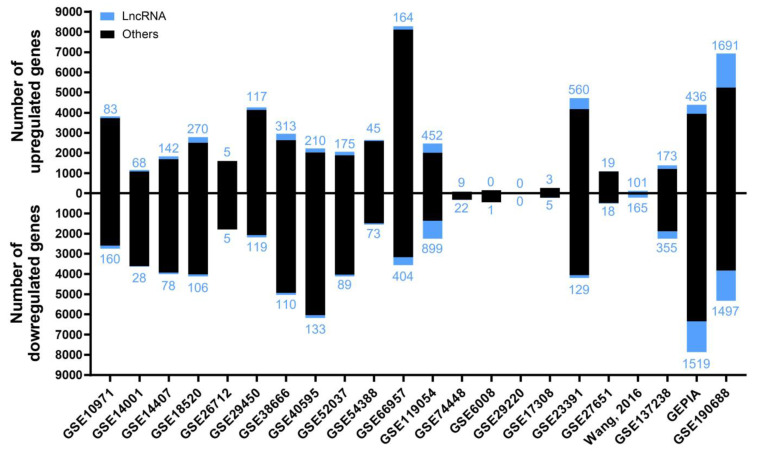
The number of deregulated genes in each selected study in the “Diagnostic” analysis. The bar graph shows the absolute frequency of upregulated (upper part) and downregulated (lower part) genes, depicting the fraction corresponding to lncRNA genes in blue at the ends of the bars.

A summary from these 22 analyses is shown in Figure 1, in which the number of up- and downregulated lncRNAs in EOC is depicted, as well as the sum of the other gene biotypes (protein-coding genes, pseudogenes, micro-RNA genes, T-cell receptor genes, immunoglobulin genes, small nuclear RNA genes, small nucleolar RNA genes, small Cajal body-specific RNA genes, ribosomal RNA genes, and ribozyme genes). The study generating the highest number of deregulated targets was GSE190688, with 6934 up- and 5325 downregulated, of which 1691 and 1497 were lncRNAs, respectively. For GSE29220, statistically significant deregulation was not identified.

**Figure 2:**
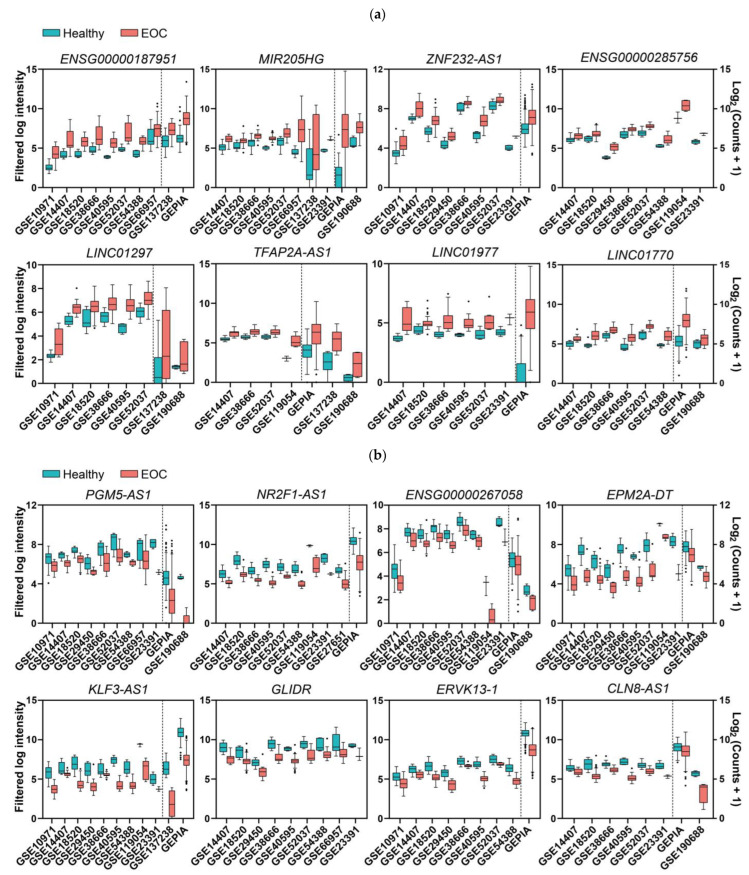
Expression levels in EOC and normal ovary tissues of the top deregulated lncRNAs that were discovered in the EOC meta-analysis. (**a**,**b**) Panels correspond to upregulated and downregulated genes, respectively. The middle line in each boxplot represents the median value, and the black dots are the outlier values. Comparisons from the dotted line to the left are from microarray studies, and comparisons from the dotted line to the right are from RNA-Seq studies, except for *GLIDR* and *ENSG00000285756*, for which all comparisons are from microarrays. *ZNF232-AS1*, *ATP2A1-AS1*, *LINC01977*, *TFAP2A-AS1*, and *NR2F1-AS1* are not represented in this figure, because only fold changes and *p*-values are available in this study [20]. All comparisons are *p* ≤ 0.01.

After obtaining the list of differentially expressed genes for each study, we carried out multiple pairwise comparisons to determine which lncRNAs were deregulated across the different cohorts with the same trend (up or down). A total of 271 lncRNAs with contradictory trends among different studies (that is, upregulated in some studies but downregulated in others) were excluded, but they are listed in the Supplementary Materials (Table S3). There were 247 upregulated and 243 downregulated lncRNAs that were statistically significant in at least three of the analyzed studies (listed in Table 1 and Table 2, respectively); the majority of which (200 and 209, respectively) were not previously related to EOC (Supplementary Materials, Table S4). The most frequently upregulated lncRNAs were *RNF157-AS1* and *BBOX1-AS1*, both in 12 different studies, whereas *MAGI2-AS3* was the most frequently downregulated lncRNA, in 15 different studies. The lncRNAs that showed significant upregulation and that were more frequently found in different cohorts, but that had not been previously related to EOC diagnosis, were *ENSG00000187951*, *MIR205HG*, *ZNF232-AS1*, *ENSG00000285756*, *LINC01297*, *TFAP2A-AS1*, *LINC01977*, and *LINC01770*, as shown in Figure 2a. The lncRNAs that were more frequently downregulated in different cohorts but that had not been previously related to EOC diagnosis were *PGM5-AS1*, *ENSG00000267058*, *EPM2A-DT*, *NR2F1-AS1*, *KLF3-AS1*, *GLIDR*, *ERVK13-3*, and *CLN8-AS1*, as shown in Figure 2b. There were several lncRNAs that we considered to be contradictory from the results that we found in the meta-analyses (Supplementary Materials, Table S3) and that were related to EOC in the literature, such as *PART1* [18] or *XIST* [19]. This fact does not invalidate the experimental evidence collected about their role in EOC, although it downplays their importance as diagnostic biomarkers for the disease, since different trends are observed in different cohorts.

Once we had obtained these two lists, we checked whether these lncRNAs were also dysregulated in 57 epithelial ovarian cancer cell lines when compared with OELE—a non-cancerous human ovarian epithelium cell line—as a criterion for further validation. We obtained raw read counts from RNA sequencing of each of these cell lines from the Cancer Cell Line Encyclopedia (CCLE), rounded them to the closest integer, and normalized them using DESeq2. After that, we sorted the 58 cell lines in ascending normalized read counts for each gene, annotating the position and quartile in which OELE was present. We considered that deregulation in patients was consistent with the cell line data when OELE occupied a position within the first quartile (lower expression) for upregulated genes or the fourth quartile (higher expression) for downregulated genes. There were 42 upregulated and 57 downregulated lncRNAs that fulfilled these criteria (in bold in Table 1 and Table 2, and reported, together with position and quartile information, in the Supplementary Materials (Table S4)). 

### 2.2. Analysis of lncRNAs with Putative Prognostic Value in EOC

**Figure 3:**
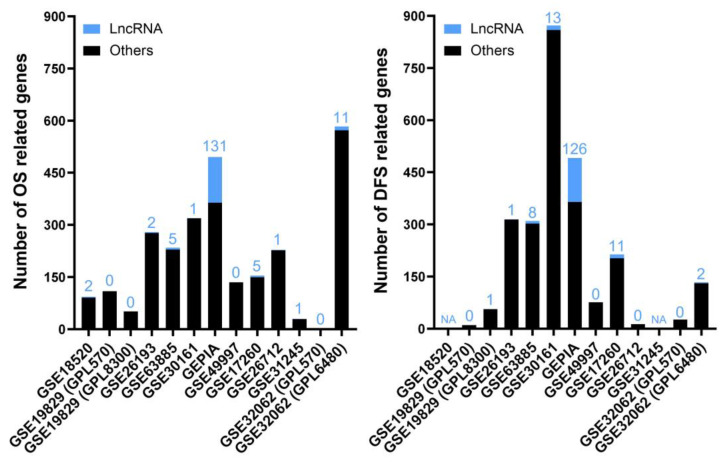
The numbers of genes affecting OS (**left**) and DFS (**right**) in each selected study for the “Prognostic” analysis. The bar graph shows the absolute frequency of genes; the portion in blue at the ends of the bars is the fraction corresponding to lncRNA genes. NA (not applicable) means that there is no patient information about DFS for those studies, whereas 0 means that there are no statistically significant genes.

This second analysis consisted of differentially expressed lncRNAs showing a significant correlation with favorable or unfavorable prognosis—meaning increased or decreased, respectively, overall survival (OS) and/or disease-free survival (DFS) periods after diagnosis or time without relapse after being treated or surgically debulked. In this category, we selected 11 studies containing information about death and relapse events over time. The metadata information of each study and the results from our meta-analysis can be found in the Supplementary Materials (Table S5). The numbers of genes resulting from the Cox proportional hazards model affecting OS and DFS for each study are depicted in Figure 3.

**Figure 4:**
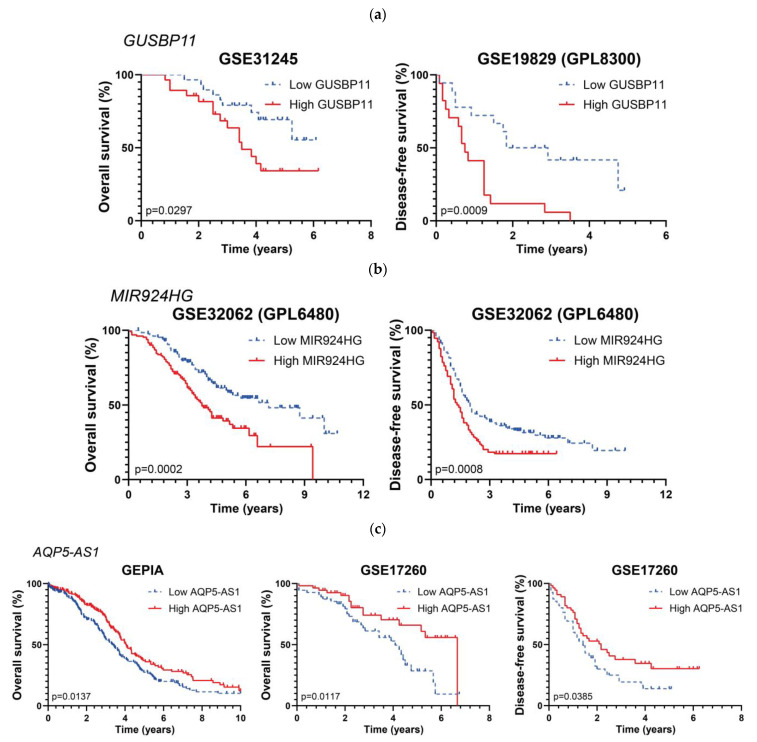
Kaplan–Meier curves for three lncRNAs affecting survival periods in EOC patients: (**a**) *GUSBP11*, (**b**), *MIR924HG*, (**c**), and *AQP5-AS1*. Graph titles indicate each corresponding study.

These analyses yielded 123 and 32 lncRNAs positively correlated with longer or shorter OS periods, respectively, as listed in Table 3. We also found 125 lncRNAs that were positively correlated with longer DFS periods and 34 lncRNAs with shorter DFS periods, as presented in Table 4. The references from the genes that were previously associated with EOC in the literature can be found in the Supplementary Materials (Table S6). When comparing the resulting gene lists from each analysis pairwise, only six lncRNAs (in bold in Table 3 and Table 4) were confirmed in two different studies: *RNF157-AS1*, *AQP5-AS1*, *CRNDE*, and *ZFAS1* in OS studies, and *MALAT1* and *SNHG8* in disease-free survival studies. However, *SNHG8* showed opposed trends in the two studies; therefore, we excluded it from the final lists of this category (but it is included in the Supplementary Materials (Table S3)). Figure 4 shows the survival periods of three selected lncRNAs.

### 2.3. Analysis of lncRNAs Deregulated in EOC Metastasis

The third analysis comprised seven different studies (summarized in the Supplementary Materials (Table S7)) containing transcriptomic information derived from the primary tumor, cancer cells from the ascitic fluid, and/or peritoneal solid metastasis samples, matched for each patient. 

**Figure 5:**
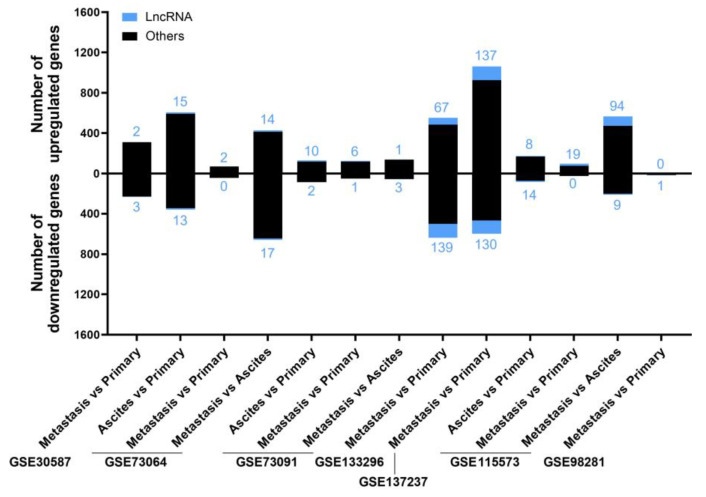
The number of deregulated genes in each comparison within the selected studies for the “Metastatic” analysis. The bar graph shows the absolute frequency of upregulated (upper part) and downregulated (lower part) genes, depicting the fraction corresponding to lncRNA genes in blue at the ends of the bars.

We analyzed the seven studies individually to look for differentially expressed lncRNA genes related to the metastatic process by comparing (i) peritoneal solid metastasis versus cancer cells from the ascitic fluid, (ii) peritoneal solid metastasis versus primary tumor, and (iii) cancer cells from the ascitic fluid versus primary tumor, using the variable “patient” as a blocking factor. A summary of the differentially expressed genes for each comparison and study is shown in Figure 5. The two studies that yielded the highest numbers of differentially expressed genes were GSE133296 and GSE137237.

After carrying out the individual analyses, we found 287 upregulated and 287 downregulated lncRNAs, of which 30 and 8, respectively, were found in two or three different studies carried out with patients, or in one study carried out with patients and differentially expressed in EOC cell lines from a metastatic origin when comparing them with those originating from primary tumors (Table 5 and Table 6). Additionally, we identified 26 lncRNAs that were upregulated or downregulated in cancer cells from ascites but did not change their levels between primary tumor and solid peritoneal metastasis, which we named “switch” (Table 7). The full lists, including references from genes previously associated with EOC, can be found in the Supplementary Materials (Table S8). Those lncRNAs whose expression was contradictory between different analyzed studies were excluded from Table S8 but included in Table S3.

**Figure 6:**
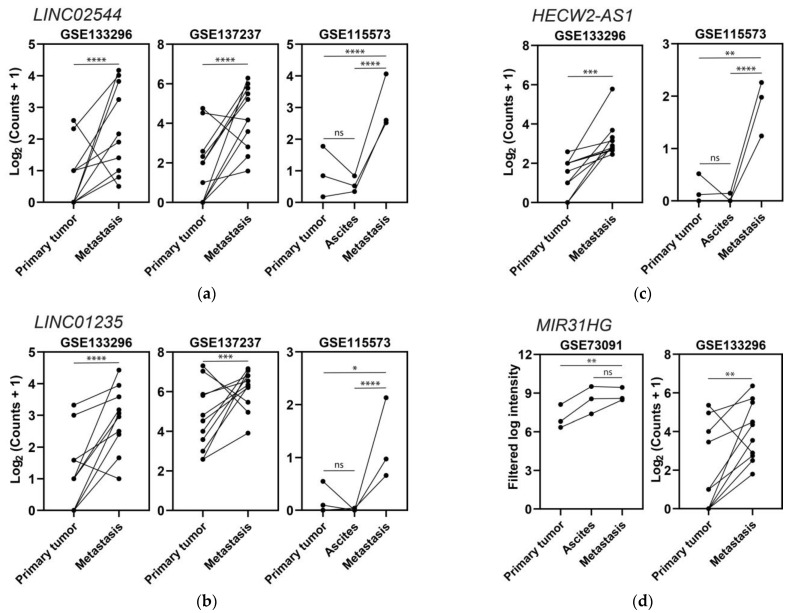
Expression levels of four lncRNAs upregulated in metastases: (**a**) *LINC02544*, (**b**) *LINC01235*, (**c**) *HECW2-AS1*, and (**d**) *MIR31HG*. Dots represent tissue samples, and lines join the samples from each patient; * *p* ≤ 0.05, ** *p* ≤ 0.01, *** *p* ≤ 0.001, **** *p* ≤ 0.0001, ns *p* > 0.05.

After obtaining these lists, we tested again whether we could correlate these results with cell lines using CCLE expression data, since there are 32 and 25 epithelial ovarian cancer cell lines that are derived from primary tumors and metastases, respectively. We first looked for differentially expressed genes between the primary and metastatic cell line groups using DESeq2 and obtained three upregulated and one downregulated (*p*-value < 0.05 and |log2 fold-change| > 0.585) lncRNAs that were present in the upregulated and downregulated lists obtained from the analyses of the patient studies. The second approach was to carry out receiver operating characteristic (ROC) curve analyses to see if the genes outlined from the patient analyses also showed differences in the comparison between the metastatic and primary tumor cell line groups. We obtained five upregulated and seven downregulated lncRNAs (area under the curve > 0.5 and *p*-value < 0.05) contained in the lists obtained from the analyses of the metastatic patient studies. The results for both DESeq2 and ROC for the metastasis category can be found in the Supplementary Materials (Table S8). In terms of the influence of lncRNAs in EOC metastasis, upregulation of *LINC02544*, *LINC01235*, *HECW2-AS1*, and *MIR31HG* in relation to this process was derived from this meta-analysis (Figure 6), and since it has not been previously found in EOC, this deserves special mention. 

### 2.4. Looking for Outstanding lncRNAs Related in Common to Diagnosis, Prognosis, and Metastasis in EOC

**Figure 7:**
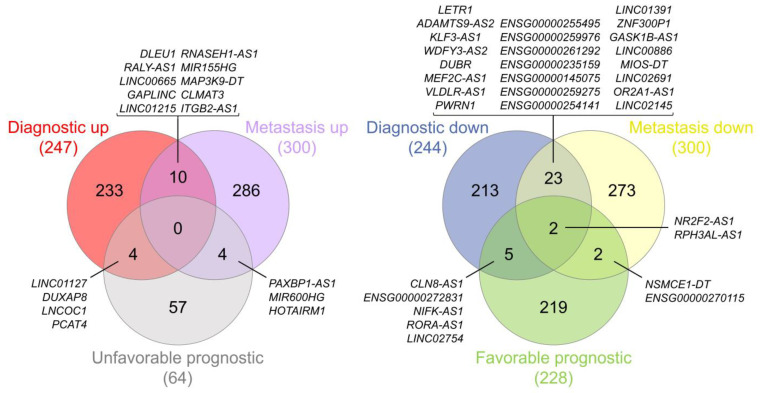
Venn diagrams representing the intersections between the final lists of the diagnostic, prognostic, and metastasis analyses—specifically, potential oncogenes (**left**) and potential tumor suppressors (**right**). Diagrams were generated using interactivenn.net.

At this point of the analysis, we decided to overlap the final lists for the first three categories to highlight the most interesting lncRNAs in terms of clinical value. We generated two different intersections, as represented in Figure 7: one comparing the upregulated lncRNAs, which represent the ones that would be putative oncogenes, and another one comparing the downregulated lncRNAs, which represent the ones that could be considered tumor suppressors.

**Figure 8:**
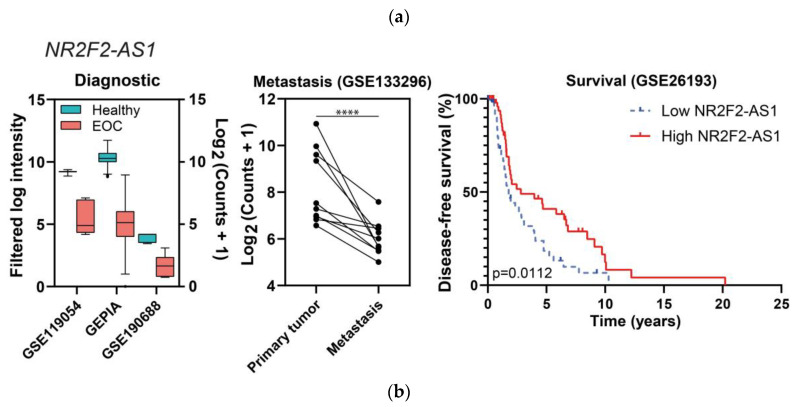

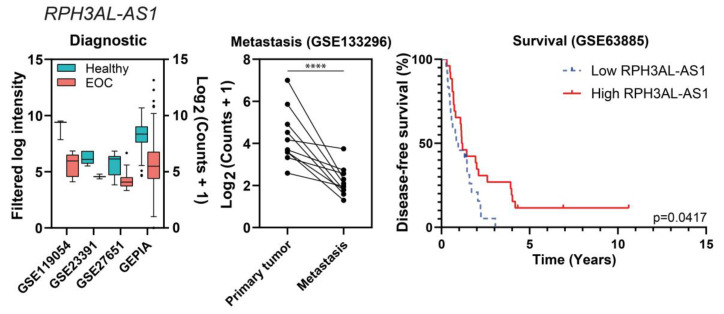
“Diagnostic” and “Metastasis” differential expression results, and survival plots of the potential tumor suppressors *NR2F2-AS1* (**a**) and *RPH3AL-AS1* (**b**). In the left panels, all of the comparisons are *p* ≤ 0.01. In the middle panels, dots represent tissue samples and lines join the three samples from each patient. In the case of *RPH3AL-AS1*, one study from the diagnostic category is not represented because raw information from [20] is not available, only fold changes and *p*-values; **** *p* ≤ 0.0001.

We only obtained results in the intersection of the three lists for the potential tumor suppressor comparison, whereas in the other comparison the intersections found were only pairwise. The lncRNAs considered to be probable tumor suppressors in EOC were *NR2F2-AS1* and *RPH3AL-AS1*, and their data from the diagnostic, metastasis, and prognostic analyses are represented in Figure 8 (see also Figure S1).

*NR2F2-AS1* and *RPH3AL-AS1* were both downregulated in EOC in comparison with healthy tissues in four independent studies (*NR2F2-AS1* was also downregulated in EOC cell lines) and in one study of metastatic tumors, correlating with a favorable prognosis (specifically, with improved disease-free survival), as shown in Figure 8. 

### 2.5. Analysis of lncRNAs with Putative Influence in Resistance to Chemotherapy

**Figure 9:**
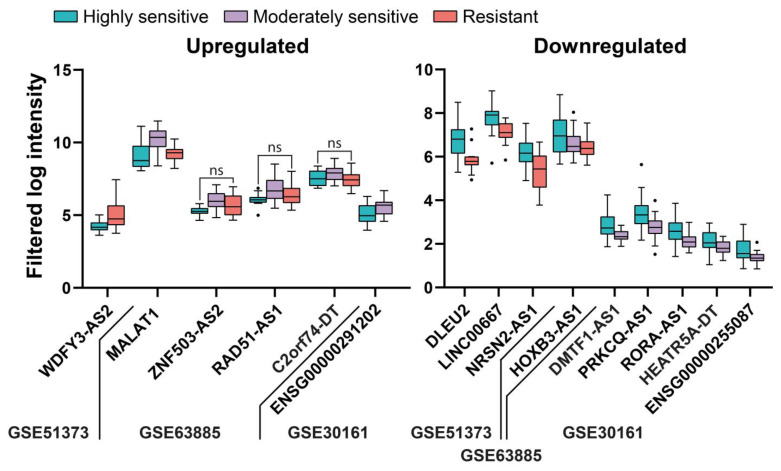
Deregulated lncRNAs in tissue from patients according to the sensitivity to standard chemotherapy. All of the comparisons are *p* < 0.05 except for those indicated with ns, indicating *p* > 0.05.

Next, we aimed to identify lncRNAs related to standard chemotherapy resistance (jointly considering cisplatin or carboplatin and/or a taxane or cyclophosphamide) by comparing expression levels from partial or non-responder and responder patients. In this case, only three studies (all microarrays) had information regarding the response to treatment, and they are listed together with the results from the individual analyses in the Supplementary Materials (Table S9). We could only find six upregulated and nine downregulated lncRNAs, as shown in Figure 9. As expected, due to the low number of deregulated genes in this category, multiple pairwise comparisons did not produce any overlap between studies. The references from previous associations between these genes and EOC can be found in the Supplementary Materials (Table S10).

Although there have been few transcriptomic studies considering the influence of lncRNAs in resistance to chemotherapy in EOC, our meta-analysis confirmed the influence of two lncRNAs previously associated with resistance to chemotherapy in EOC—*WDFY3-AS2* [21], and *MALAT1* [22]—as well as revealing the influence of other lncRNAs that have been associated with EOC but not with chemoresistance until now, i.e., *LINC00667* [12], *NRSN2-AS1* [23], and *RAD51-AS1* [24]. Others had not been associated with EOC but had been associated with other cancers, i.e., *DLEU2* with endometrial [25] and prostate cancers [26], *LINC00667* with breast cancer [27], and *PRKCQ-AS1* with multiple myeloma [28].

### 2.6. Analysis of lncRNAs with a Putative Specific Value Associated with Histological EOC Subtypes

Epithelial ovarian cancer can be further classified into five subtypes according to histological structure, mutations in certain tumor suppressors or proto-oncogenes, chemosensitivity, spreading behavior, and patient prognosis [29]. These five subtypes and their relative frequencies are high-grade serous (HGSOC, 70%), low-grade serous (LGSOC, >5%), endometrioid (ENOC, 10%), clear cell (CCOC, 10%), and mucinous (MOC, 3%) [29]. As there were six studies with the subtype information available (Supplementary Materials, Table S11), in the last analysis, we looked for lncRNAs whose expression was specific for each EOC subtype.

**Figure 10:**
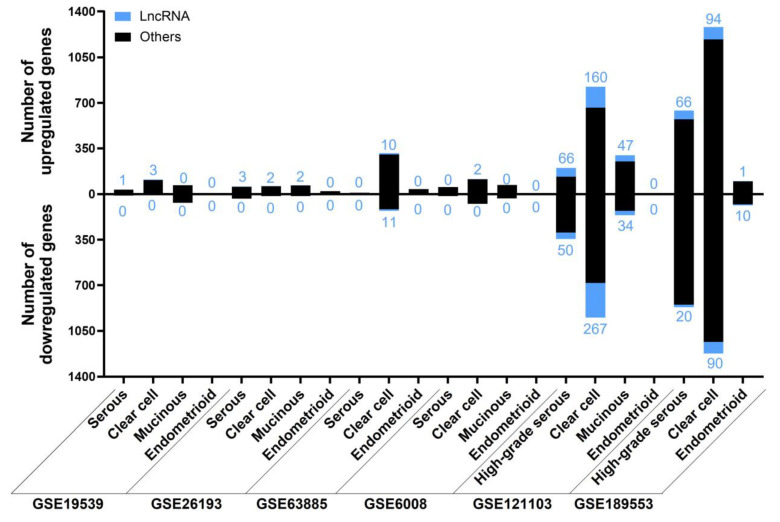
The number of deregulated genes in each selected study for the “Subtype” analysis. The bar graph shows the absolute frequency of upregulated (upper part) and downregulated (lower part) genes, depicting the fraction corresponding to lncRNA genes in blue at the ends of the bars.

The results of each analysis for the subtype category are contained in the Supplementary Materials (Table S11) and summarized in Figure 10. Regarding HGSOC and LGSOC, we did not consider LGSOC independently, because there was only information about this subtype in two studies (GSE14001 and GSE26751), in which there were HGSOC and healthy individuals and no other subtypes to compare with. We did not find any ambiguous lncRNAs within any of the subtypes across different studies. Those lncRNAs that were present in two or three studies of HGSOC or CCOC are listed in Table 8 and Table 9, respectively. The full list of deregulated lncRNAs according to each subtype can be found in the Supplementary Materials (Table S12).

**Figure 11:**
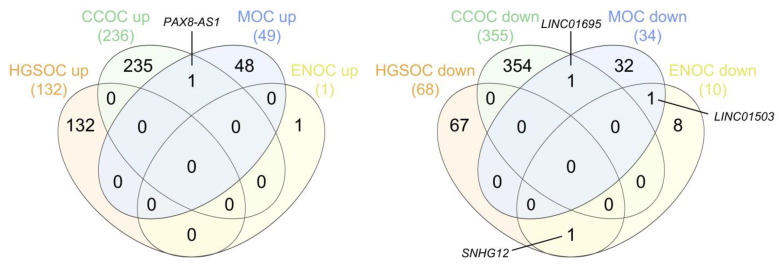
Venn diagrams representing the intersections between the final lists of the upregulated (**left**) and downregulated (**right**) subtype-specific lncRNAs. HGSOC, high-grade serous ovarian carcinoma; CCOC, clear cell ovarian carcinoma; MOC, mucinous ovarian carcinoma; ENOC, endometrioid ovarian carcinoma. Diagrams were generated using interactivenn.net.

We performed two different multiple pairwise comparisons (up- and downregulated lncRNAs, separately) between the lists drawn for each subtype to identify true subtype-specific lncRNAs and exclude those present in more than one subtype. The results of these overlaps are shown in Figure 11. We found an a priori unexpected specificity between lncRNAs and histological subtypes of EOC. Surprisingly, only 1 out of the 418 upregulated and 3 out of the 467 downregulated lncRNAs in any subtype were deregulated in the same way in two different subtypes (included in the Supplementary Materials (Table S3)), meaning that the remaining lncRNAs are potentially subtype-specific.

## 3. Discussion

The lack of early detection methods for EOC and its consequent late diagnosis result in dramatic high mortality. We aimed to update the significance of putative lncRNA-based biomarkers in EOC in light of available studies combining gene expression and clinical data. 

As the first objective of our analysis, we sought to identify differentially expressed lncRNAs (either upregulated or downregulated) in EOC compared with healthy samples that could be identified as significant candidates for future experimental analysis and translation to clinical practice as diagnostic biomarkers. Data from 22 different cohorts of patients were included in this analysis. Indeed, lncRNA expression in these cohorts had been previously analyzed in some cases, but not all. The reanalysis of all of these available data revealed worthwhile information because it allowed us to bring to light available, but not evident, information. We found 247 upregulated and 243 downregulated lncRNAs (listed in Table 1 and Table 2, respectively), 200 and 209 of which were not previously related to EOC, respectively. The most frequently upregulated lncRNAs were *RNF157-AS1* and *BBOX1-AS1*, both in 12 different studies, whereas *MAGI2-AS3* was the most frequently downregulated lncRNA, in 15 different studies. Validation of these novel EOC-related lncRNAs is supported by three pieces of evidence: First, the meta-analysis, applied to protein-coding genes across the same studies, rendered a group of genes that are well-known EOC markers as significantly deregulated, including *CP* [30], *CD24* [31], and *INAVA* [14]—upregulated in 18, 17, and 16 studies, respectively—as well as *AOX1* [14] and *PDGFD* [32], downregulated in 16 and 13 studies, respectively. Second, our analysis also found other lncRNAs previously related to EOC (cited in the Supplementary Materials (Table S4)). These include, among others, *RNF157-AS1* [33] and *UCA1* [34], which interact with proteins to regulate transcription; *BBOX1-AS1* [35], *DUXAP8* [36], *LINC01503* [37], *HAGLR* (as *HOXD-AS1*) [38], *LINC00665* [39], and *HAGLROS* [40], which act as competing endogenous RNAs (ceRNAs) with miRNAs, affecting gene expression at the post-transcriptional level; and *MAGI2-AS2* [41], *HAND2-AS1* [42], *ZNF300P1* [43], and *HYMAI* [44], whose downregulation in EOC is explained by hypermethylation of the promoter. The third piece of evidence for the validation of our meta-analysis comes from the fact that 99 deregulated lncRNAs in EOC patients were also deregulated in EOC cell lines (Supplementary Materials, Table S4). The lncRNAs that showed significant upregulation, and which were more frequently found in different cohorts, but that had not been previously related to EOC diagnosis, were *ENSG00000187951*, *MIR205HG*, *ZNF232-AS1*, *ENSG00000285756*, *LINC01297*, *TFAP2A-AS1*, *LINC01977*, and *LINC01770*, as shown in Figure 2a. Some of them have been previously related to other cancer types. *MIR205HG* is the host gene for the microRNA miR-205 but, although originating from the same primary transcript through alternative splicing, its lncRNA and miRNA are functionally independent [45,46,47]. *MIR205HG* promotes lung squamous cell carcinoma [48], osteosarcoma [49], melanoma [50], cervical cancer [51,52], head and neck squamous carcinoma [46], and esophageal squamous carcinoma [53]; however, in esophageal adenocarcinoma, it is downregulated and hinders *HNRNPA0* mRNA translation by interacting with *LIN28A* [54] and affecting the Hedgehog pathway [55]. *MIR205HG* also acts in physiological processes, such as in embryogenesis by regulating the transcription of Pit1, Zbtb20, prolactin, and growth hormone in the anterior pituitary in mouse models [47]; or in cell fate by preventing luminal differentiation of human prostate basal cells through the interferon pathway by forming a DNA:RNA triplex in the *Alu* regulatory elements in the proximal promoter of target genes [45,56]. *LINC01770* is upregulated in endometrial cancer in comparison with endometrial dysplasia tissues [57]. *LINC01977* is upregulated and correlated with poor prognosis in lung adenocarcinoma [58] and breast cancer [59]; in lung adenocarcinoma, TGF-β derived from infiltrated tumor-associated macrophages (TAM2) activates SMAD3, which binds *LINC01977* to induce its nuclear transport, where it upregulates transcription by simultaneously binding the promoter and super-enhancer, facilitating the interaction between SMAD3 and CBP/P300 to activate *ZEB1* transcription [58]. In breast cancer, *LINC01977* expression is also correlated with chemoresistance to doxorubicin by targeting the miR-212-3p/*GOLM1* axis [59]. *LINC01297* is upregulated in estrogen receptor (ER)-positive breast cancer, in comparison with ER-negative breast cancer [60] and lung adenocarcinoma [61]; there is also a positive correlation between *LINC01297* and the expression of its nearby gene *LINC01296*, which acts as an oncogene in bladder cancer [62]. Conversely, *ENSG00000285756* and *TFAP2A-AS1*, which were EOC-upregulated lncRNAs in our analysis, are downregulated in other cancer types. *ENSG00000285756* is downregulated in cervical cancer [63], and *TFAP2A-AS1* is downregulated and correlated with a good prognosis in breast cancer, acting in vitro as a tumor suppressor by sponging miR-933 to modulate *SMAD2* mRNA stability [64]; *TFAP2A-AS1* is also transcriptionally activated by KLF15 and inhibits the proliferation and migration of gastric cancer cells by sponging miR-3657 to regulate *NISCH* mRNA stability [65]. The lncRNAs that were more frequently downregulated in different cohorts, but that had not been previously related to EOC diagnosis, were *PGM5-AS1*, *ENSG00000267058*, *EPM2A-DT*, *NR2F1-AS1*, *KLF3-AS1*, *GLIDR*, *ERVK13-3*, and *CLN8-AS1*, as shown in Figure 2b. *PGM5-AS1* (*ENSG00000224958*) is downregulated in both EOC patients and EOC cell lines; it is also downregulated and negatively correlated with oxaliplatin resistance in colorectal cancer [66] but, on the other hand, it is upregulated and sponges miR-140-5p to prevent *FBN1* mRNA degradation in osteosarcoma [67]. *KLF3-AS1* is downregulated in esophageal squamous cell carcinoma stem cells, promoting cell migration and invasion by being unable to sponge miR-185-5p, which induces *KLF3* mRNA degradation, thereby preventing transcriptional repression of *SOX2* and *OCT4* by KLF3 [68], and acts as a competing endogenous tumor suppressor RNA in gastric cancer [69] and osteosarcoma [70]. Conversely, several lncRNAs that are downregulated in EOC are upregulated and stimulate cancer progression in other tissues. *NR2F1-AS1* is upregulated in non-small cell lung [71], thyroid [72], pancreatic [73], and hepatocellular [74] cancers; in pancreatic cancer, it is induced by hypoxia, its expression is positively correlated with the expression of its sense gene *NR2F1*, and they both trigger the AKT and mTOR pathways, promoting proliferation, migration, and invasion [75]. *NR2F1-AS1* is also upregulated in dormant breast cancer stem-like cells and increases tumor dissemination by recruiting PTBP1 to the mRNA of its sense gene (*NR2F1*), promoting its translation so that NR2F1 represses *ΔNp63* transcription [76]. *GLIDR* is upregulated and promotes glioma [77], lung [78], and prostate [79] cancers by acting as competing endogenous RNAs for miRNAs. *ERVK13-3* is upregulated in osteosarcoma [80].

Our second objective was to find differentially expressed lncRNAs showing a significant correlation with favorable or unfavorable prognosis. From our initial selection of studies from 46 different cohorts, only 11 contained information about death and relapse events over time. The analysis of these data rendered a limited amount of lncRNAs whose differential expression could be related to prognosis. Among them, high expression of *GUSBP11* and *MIR924HG* (underlined in Table 3 and Table 4) was positively correlated with shorter OS and DFS, meaning a negative prognosis. In accordance with our data for EOC, high expression levels of *GUSBP11* are predicted to bind miR-22-3p to avoid CCN2A mRNA degradation, correlating to poor overall survival in hepatocellular carcinoma patients [81]. Conversely, *GUSBP11* expression correlates with better prognosis in head and neck squamous cell carcinoma [82], bladder cancer [83], papillary renal cell carcinoma [84], and pancreatic adenocarcinoma [85]. Regarding *MIR924HG*, there is no information about it in the literature. However, miR-924, which is hosted in the *MIR924HG* locus, acts as a tumor suppressor in non-small-cell lung carcinoma [86] and hepatocellular carcinoma [87]. In our analysis, *AQP5-AS1* was associated with a more favorable prognosis due to its correlation with longer survival periods in different studies. There is no information regarding the antisense lncRNA *AQP5-AS1* in the literature, but the sense transcript encodes the protein *AQP5*, which is overexpressed in OC tissues [88] and promotes proliferation and migration in OC [89]; however, contradictory data can also be found, since high AQP5 expression is correlated with a better prognosis in OC patients [90].

The third objective was the identification of differentially expressed lncRNAs associated with metastasis in EOC. Seven studies included samples that allowed comparisons of (i) peritoneal solid metastasis vs. cancer cells from the ascitic fluid, (ii) peritoneal solid metastasis vs. primary tumor, and (iii) cancer cells from the ascitic fluid vs. primary tumor. Upregulation of *LINC02544*, *LINC01235*, *HECW2-AS1*, and *MIR31HG* in relation to this process was found from this meta-analysis and, since it has not been previously found in EOC, deserves special mention. Previous data about the influence of these lncRNAs in invasion or metastasis and unfavorable prognosis can be found in other cancers and reinforce our findings. *LINC02544* is overexpressed in lung squamous cell carcinoma patients with lymph node metastasis, and it promotes proliferation, migration, and invasion in vitro by sponging miR-138-5p—a potential target of E2F3 [91]; increased expression of *LINC02544* has also been related to in vitro invasion and unfavorable prognosis in breast cancer [92]. *LINC01235* is upregulated in gastric cancer patients versus healthy individuals and metastatic versus non-metastatic gastric cancer patients, promoting migration, invasion, and EMT, as well as negatively affecting prognosis [93,94]. *MIR31HG* has been associated with unfavorable prognosis in gastric cancer [95], lung adenocarcinoma [96], head and neck squamous cell carcinoma [97,98], colorectal cancer [99], and non-small cell lung carcinoma [100].

The intersection of differentially expressed lncRNAs obtained in the three previous analyses (diagnosis, prognosis, and metastasis) highlighted the importance of two lncRNAs (*NR2F2-AS1* and *RPH3AL-AS1*) in EOC. A priori, we did not expect a large overlap between lncRNAs useful in diagnosis and prognosis, since lncRNAs discovered in each category may be regulating different phases and functions of oncogenesis. A greater overlap exists between metastasis and prognosis, since the functional relationship between metastasis and prognosis is very straightforward, and the presence of metastasis is usually associated with shorter survival times. *NR2F2-AS1* is upregulated in several malignancies, such as non-small cell lung cancer, clear cell renal cell carcinoma, and prostate, cervical, nasopharynx, and esophageal cancers, being considered an oncogene [101], contrary to what we observed in EOC. Its gene product NR2F2 or COUP-TFII is highly expressed in the ovarian stroma and is negligible in the ovarian epithelium, although NR2F2 is downregulated at the mRNA level in OC tissues. NR2F2 expression increases in the epithelial component and is associated with shorter periods before recurrence [102]. NR2F2 acts as an oncogene in other cancers, such as renal, prostate, or breast cancers [103]. *RPH3AL-AS1* is located within the cytoband 17p13.3, and its downregulation in EOC could be due to the high deletion frequency of this chromosomal region in OC patients [104,105].

Next, as a fourth objective, we aimed to identify lncRNAs related to standard chemotherapy resistance. Among the studies considered in this meta-analysis, there were only three studies containing information that allowed us to study this correlation. Although the results from our meta-analysis confirm the influence of two lncRNAs previously associated with resistance to chemotherapy in EOC (*WDFY3-AS2* [21] and *MALAT1* [22]) and some new lncRNAs that were also detected, more studies are needed to validate these results. 

Epithelial ovarian cancer can be further classified into five subtypes according to its histological structure: high-grade serous, low-grade serous, endometrioid, clear cell, and mucinous [29]. We found in our meta-analysis that the differential expression of lncRNAs is highly specific for the different subtypes, which can be used for diagnostic purposes.

One of the most interesting characteristics of lncRNAs is that they can be detected in tumor-derived small extracellular vesicles, as well as free molecules or protein-associated complexes circulating in the blood. The fact that circulating levels of some lncRNAs in serum or plasma samples can correlate to those found in tumor tissue increases their importance as biomarkers in liquid biopsy for EOC, with ongoing clinical trials [5]. There are examples of some lncRNAs identified in our meta-analysis that have been previously found in liquid biopsies. *HAND2-AS1*—which is downregulated in EOC tumors, as confirmed in 13 studies (Table 2)—had been also detected in blood plasma from triple-negative breast cancer patients [106]. The expression of *SP2-AS1*, which was downregulated in eight studies (Table 2), was previously detected in blood and associated with the risk of endometriosis and ENOC [107]. *UCA1*, which was upregulated in 10 EOC studies (Table 2), also showed increased levels in serum-derived exosomes from cisplatin-resistant OC patients [108] and plasma from colorectal cancer patients [109]. *PGM5-AS1*, downregulated in tumor tissues from 12 EOC studies (Table 2), was also downregulated in plasma from colorectal cancer patients [109]. The lncRNA *ESRG*, upregulated in eight EOC studies (Table 2), was detected in exosomes present in effusion supernatants from HGSOC [110]. *GUSBP11*, which was correlated with a poor prognosis in EOC (Table 3 and Table 4, and Figure 4), was upregulated in plasma from gastric cancer patients in comparison with healthy individuals [111].

Finally, it is also important to remark that EOC is intrinsically associated with late diagnosis and, therefore, most of the studied samples included in this meta-analysis come from advanced stages of the disease, which implies that early-stage samples are underrepresented. Studies with larger cohort sizes and increased representation of samples from the initial stages of EOC, along with the use of sensitive, normalized, universally standardized, and reproducible techniques to detect circulating lncRNAs, are still needed to make possible the translation of these findings into the gynecological clinical setting.

## 4. Materials and Methods

### 4.1. Selection of Suitable Gene Expression Datasets for Meta-Analysis

Microarray- and bulk RNA-Seq-based gene expression profiling studies for ovarian cancer were identified in PubMed and the Gene Expression Omnibus (GEO—https://www.ncbi.nlm.nih.gov/geo/ accessed on 15 January 2023) [112]. The search terms included “ovarian cancer” AND (“microarray” OR “RNA-Seq”) AND “patients”. Eligible studies and datasets had to fulfill the following requirements: (i) include case and control human studies, (ii) perform transcriptomic analyses, and (iii) have available raw and/or processed microarray or RNA-Seq data. Studies were not considered if they were (i) letters, abstracts, and human case reports, i.e., not full and original research studies; (ii) studies based only on cell lines as a model of study; (iii) RT-qPCR-based studies only; (iv) studies neglecting ncRNAs—specifically lncRNAs; or (v) only focused on stromal or germinal ovarian cancer.

### 4.2. Data Extraction and Processing

Microarray intensity files (CEL or text), probe information tables, and RNA-Seq read count tables, along with the experimental metadata included in the series matrix files, were downloaded from the GEO Accession Display for each selected dataset, whereas FASTQ files were downloaded from the European Nucleotide Archive (ENA) browser. It is worth noting that we only considered genes with Ensembl IDs, as their GENCODE annotation is manually supervised and, therefore, updated and reliable. Because of this, some lncRNAs with only NCBI Gene IDs were not considered, although they were perhaps still interesting for EOC; thus, we may have underestimated our results for the sake of a more confident annotation. The version of the annotation was Ensembl 108 (GRCh38.p13).

#### 4.2.1. Microarray

Microarray data were processed using BRB-ArrayTools (version 4.6.2), developed by Dr. Richard Simon (Biometric Research Program, National Cancer of Institute, BeThesda, Rockville, MA, USA) and the BRB-ArrayTools Development Team. Affymetrix CEL files were imported with the Data Import Wizard option, using the JustRMA normalization method and standard Affymetrix probe set IDs. The text files were imported using the General Format Importer option, adjusting the red and green thresholds to 10 and 100, respectively, and the background intensity and spot flag information were considered when available. Regardless of the importing option, the experiment descriptor files created from the corresponding series matrix files were also imported, and in all cases the option “Average the replicate spots within an array” was selected. Affymetrix Human Genome U133 Plus “1.0” and 2.0 arrays were annotated with chip-specific Bioconductor packages, whereas the rest were annotated using information included in the intensity file or the probe information table, both resulting in EntrezId, UniGene ID, and/or GenBank Nucleotide Accession Number lists. These IDs were converted to Ensembl Gene IDs using bioDBnet [113], unified in one list, and annotated by merging with the human genome information (hg38, GRCh38.p13) using RStudio (v4.1.0)’s merge function from the data.table package. 

#### 4.2.2. RNA-Seq

RNA-Seq data were processed using RStudio (version 2022.12.0 Build 353), R (version 4.1.0), Bioconductor (version 3.14), and DESeq2 (version 1.34). For GSE190688, GSE98281, and GSE115573 studies, read count files were not available; hence, after concatenating the GSE115573 files belonging to each sample, we mapped the three of them and quantified the reads using Kallisto [114] against human (hg38) cDNA and ncRNA transcriptomes obtained from Ensembl FTP (January 2023). After merging both cDNA and ncRNA abundance files, they were imported into RStudio using tximport and implemented into DESeq2 using the function DESeqDataSetFromTximport. For the rest of the cases, read counts for each Ensembl Gene ID and sample were imported to R in a single file, including the experimental design. Gene filtering and normalization were carried out using DESeq2. In the case of GSE189553, read counts were rounded to the closest integer so that they could be analyzed by DESeq2.

When using datasets from Gene Expression Profiling Interactive Analysis [115], gene expression profiling was compared between ovarian cancer patients from the Cancer Genome Atlas (TCGA) and normal ovarian tissues from Genotype-Tissue Expression (GTEx). Specifically, in the “FUNCTIONS”, “Expression Analysis”, and “Differential Genes” tab, the “OV” dataset, which corresponds to ovarian adenocarcinoma, was selected. Moreover, from GEPIA2, the top differential genes affecting prognostic variables were downloaded. Specifically, in the “FUNCTIONS”, “Survival Analysis”, and “Most Differential Survival Genes” tab, the “OV” dataset was again selected. Additionally, GTEx normal ovary read counts were downloaded from https://gtexportal.org/home/datasets (accessed on 15 January 2023), and TCGA ovarian adenocarcinoma read counts and raw survival information were downloaded from UCSC Xena (https://xenabrowser.net/datapages/ (accessed on 15 January 2023)).

Cell line RNA-Seq read count data generated by RNA-Seq by Expectation-Maximization (RSEM), along with metadata related to the histological subtype and metastatic or non-metastatic origin of the cell lines, were downloaded from the Cancer Cell Line Encyclopedia [116]. We filtered the information from 57 epithelial ovarian cancer cell lines and 1 immortalized, non-cancerous ovarian epithelium cell line (OELE). There are 74 cell lines derived from the ovaries, but COLO704, HEY, OVMIU, DOV13, OC315, JHOS3, OVCA420, and OVCA433 do not have transcriptomic information available, and OVCAR5, HSKTC, SNU840, KGN, PA1, COV434, BIN67, and SCCOHT1 were not considered in our meta-analysis because they are not models of epithelial ovarian cancer. RSEM counts were rounded to the closest integer and then normalized using DESeq2.

### 4.3. Data Analysis

Four different types of comparisons were carried out to identify differentially expressed lncRNA genes in EOC: (I) “Diagnostic category”—cancer samples versus normal samples; (II) “Metastasis category”—peritoneal metastasis versus primary tumor, peritoneal metastasis versus cancer cells from ascites or effusion, or cancer cells from ascites or effusion versus primary tumor; (III) “Drug resistance category”—resistant versus sensitive or partial responders versus complete responders; and (IV) “Subtype category”—samples with one histological subtype versus the remaining ones. An additional comparison was carried out between metastasis-derived cell lines and primary tumor-derived cell lines using data from cell line transcriptomes.

#### 4.3.1. Differential Expression in Microarray

To identify differentially expressed genes between ovarian cancer patients and women free of the disease, class comparisons between groups of arrays were carried out using the function “Class comparison”. Two-sample *t*-tests were used, setting the significance threshold of univariate tests as 0.01, assuming (when possible) a random variance model, and blocking by patient or sample (when matched samples were available).

#### 4.3.2. Differential Expression in RNA-Seq

Differential expression was run with DESeq2 and, finally, annotated to the human genome hg38, as previously described for microarray data. The false discovery rate (FDR) cutoff was set as 0.05 and |Log2FC| as 0.585, blocking by patient or sample when matched samples were available. In the case of GEPIA2, the differential gene expression was calculated using the LIMMA method, with the |Log2FC| cutoff set as 0.585 and the q-value cutoff set as 0.01, selecting both overexpressed and underexpressed genes.

#### 4.3.3. Differential Survival in Microarray

Overall survival (OS) and disease-free survival (DFS) gene lists were generated using BRB-ArrayTools “Survival analysis” and “Genes affecting survival”. This option carries out Cox proportional hazards model (Wald statistic) univariate tests, and the *p*-value was set to 0.01.

#### 4.3.4. Differential Survival in RNA-Seq (GEPIA2)

OS and DFS gene lists were generated based on gene expression by applying the Log-rank test (Mantel-Cox test). The OV TCGA cohort threshold between the “high” and “low” groups was set to the median, the confidence interval to 95%, and the *p*-value to 0.01.

### 4.4. Pairwise lncRNA Analysis

Significantly dysregulated lncRNAs with Ensembl IDs from microarray or RNA-Seq studies were selected and separated into “upregulated” or “downregulated” lists, respectively, for each individual study according to their fold change in the previously described comparisons.

All possible pairwise comparisons between studies of each category were carried out within the “upregulated” and “downregulated” lists, separately, using the Excel match function between Ensembl Gene IDs. As an exclusion criterion for all of the categories, genes that had contradictory trends in different studies were considered to be ambiguous and were discarded. For the “Diagnostic category”, only lncRNA genes that were differentially expressed in at least three independent studies were selected. For the “Survival category”, lncRNAs were separated into those positively correlated with favorable patient prognosis or unfavorable prognosis. Finally, selected lncRNA genes for each category were further compared pairwise with the remaining categories, again using the Excel match function (version 2305).

### 4.5. ROC Analysis

Receiver operating characteristic (ROC) curve analysis was performed using easyROC (version 1.3.1.) (http://www.biosoft.hacettepe.edu.tr/easyROC/ (accessed on 15 January 2023)) [117].

### 4.6. Figures and Venn Diagrams

Figures were drown with graphpad prism version 8.0.2.Venn diagrams were generated using InteractiVenn (http://www.interactivenn.net/# (accessed on 15 January 2023) [118]).

### 4.7. Nomenclature

Novel transcripts that do not have yet a gene symbol are identified by their Ensembl Gene ID.

## 5. Conclusions

The study of lncRNAs in cancer is an emerging field, and according to GENCODE Release 43 [119] the number of known lncRNA genes in the human genome so far is 19,928, which is slightly higher than the 19,393 protein-coding genes. Despite this large number, only a small proportion of lncRNAs have been associated with EOC, and with this study we have increased this number, with 1631 new lncRNA genes. This effort has produced valuable information to take into account in future projects, since specific lncRNAs are associated with EOC for diagnosis (*ENSG00000187951*, *MIR205HG*, *ZNF232-AS1*, *ENSG00000285756*, *LINC01297*, *TFAP2A-AS1*, *LINC01977*, *LINC01770*, which are upregulated; and *PGM5-AS1*, *ENSG00000267058*, *EPM2A-DT*, *NR2F1-AS1*, *KLF3-AS1*, *GLIDR*, *ERVK13-3*, *CLN8-AS1*, which are downregulated), prognosis (*GUSBP11*, *MIR924HG*, unfavorable; and *AQP5-AS1*, favorable), and metastasis (*LINC02544*, *LINC01235*, *HECW2-AS1*, and *MIR31HG*). Furthermore, the differential expression of lncRNAs is highly specific to the different subtypes, which can be used for diagnosis purposes.

## Figures and Tables

**Table 1:** Upregulated lncRNAs in ovarian cancer tissue in at least three different transcriptomic studies from the “Diagnostic” analysis. For genes in bold, the position of the non-cancerous cell line OELE matches the first quartile when sorting the cell lines in ascending normalized counts.

Number of Studies	Gene Count	Gene Name
**12**	2	*RNF157-AS1*, *BBOX1-AS1*
**11**	1	*DUXAP8*
**10**	6	*ATP2A1-AS1*, ***ENSG00000187951***, *FOXP4-AS1*, *MIR205HG*, ***UCA1***, *ZNF232-AS1*
**9**	2	***LINC00664***, ***LINC01503***
**8**	10	***ENSG00000285756***, *ESRG*, *HAGLR*, *LINC00665*, *LINC01297*, *LINC01770*, ***LINC01977***, *PRKCQ-AS1*, *TFAP2A-AS1*, *TIAM1-AS1*
**7**	8	*ASH1L-AS1*, *HAGLROS*, *LINC00839*, *LINC01135*, *LINC01558*, *MRPL20-AS1*, ***SPINT1-AS1***, *VPS13B-DT*
**6**	11	*C10orf95-AS1*, ***DLX6-AS1***, *ENSG00000286546*, *FZD10-AS1*, *KLHDC7B-DT*, *LINC03014*, *NCK1-DT*, *PCAT6*, ***PP7080***, *RNASEH1-AS1*, *TDRKH-AS1*
**5**	25	*CCDC140*, *CDKN2B-AS1*, *DLEU1*, *DPP3-DT*, *ELFN1-AS1*, *WDR35-DT*, ***PPP1R3B-DT***, *ENSG00000256802*, *KDM7A-DT*, *KIAA1614-AS1*, *KIF25-AS1*, *LBX2-AS1*, *LINC00853*, *LINC00884*, *LINC01142*, *LINC01215*, *LINC01532*, *LINC02159*, *LINC02387*, *LYRM4-AS1*, *MIR155HG*, *MYCNOS*, ***OBSCN-AS1***, *TLR8-AS1*, ***TNFRSF10A-DT***
**4**	46	*ATP11A-AS1*, *CLMAT3*, ***DSP-AS1***, ***ENSG00000231119***, *ENSG00000260418*, *ENSG00000267665*, *EPHA1-AS1*, *EPIC1*, ***EXOSC10-AS1***, *GAPLINC*, *ITGB2-AS1*, *LAMA5-AS1*, *LINC00943*, *LINC00944*, ***LINC00958***, *LINC01224*, *LINC01271*, ***LINC01315***, *LINC01333*, *LINC01342*, *LINC01356*, *LINC01362*, *LINC01410*, *LINC01545*, *LINC01547*, *LINC02041*, *LINC02043*, *LINC02492*, *LINC03011*, *LINGO1-AS1*, *MINCR*, ***MIR1915HG***, *MIR200CHG*, *NAV2-AS5*, *PCAT4*, ***PPP1R14B-AS1***, *PPP1R26-AS1*, *PRRT3-AS1*, *PRRX2-AS1*, *PSORS1C3*, *RALY-AS1*, *SLC2A1-DT*, *SNHG4*, *ZNF503-AS1*, *ZNF687-AS1*, *ENSG00000215022*
**3**	136	*ANKRD44-IT1*, ***BISPR***, *BRWD1-AS2*, *C6orf99*, ***C8orf31***, *CA3-AS1*, *CARD8-AS1*, *CASC9*, *CSMD2-AS1*, *CT69*, *CTB-178M22.2*, *DHCR24-DT*, *DHX35-DT*, *EGFLAM-AS4*, *ENSG00000224504*, *ENSG00000226527*, *ENSG00000259540*, ***ENSG00000260912***, *ENSG00000261095*, ***ENSG00000261924***, *ENSG00000273523*, *ENSG00000289161*, *ENSG00000290993*, *ENSG00000291232*, *FAM151B-DT*, *FZD4-DT*, *GNAS-AS1*, ***GPRC5D-AS1***, *HMMR-AS1*, *IQCF5-AS1*, *KANSL1-AS1*, *KCNMB2-AS1*, ***LCAL1***, ***LCMT1-AS2***, *LINC00308*, *LINC00354*, *LINC00461*, *LINC00513*, *LINC00589*, *LINC00620*, *LINC00656*, *LINC00668*, *LINC00858*, *LINC00868*, *LINC00896*, *LINC00907*, *LINC01049*, *LINC01111*, ***LINC01123***, ***LINC01127***, *LINC01144*, *LINC01512*, *LINC01737*, *LINC01812*, ***LINC01833***, *LINC01904*, *LINC02064*, *LINC02152*, *LINC02223*, *LINC02240*, *LINC02257*, *LINC02517*, *LINC02580*, *LINC02712*, *LINC02837*, *LNCOC1*, *LZTS1-AS1*, ***MAP3K9-DT***, *MAPT-AS1*, *MIR3681HG*, *MIR3945HG*, *MIR4458HG*, *MRPL20-DT*, *NDUFB2-AS1*, ***NEBL-AS1***, ***NINJ2-AS1***, *NUP50-DT*, *NUTM2A-AS1*, ***OGFRP1***, *PARTICL*, *PIK3CD-AS2*, *POU2F2-AS1*, *PSLNR*, *PTPRN2-AS1*, *RAPGEF4-AS1*, *RFX5-AS1*, ***RHPN1-AS1***, *RNASE11-AS1*, ***SAMD12-AS1***, *SCIRT*, *SLC8A1-AS1*, *STAU2-AS1*, *TIPARP-AS1*, *TRPM2-AS*, *TYMSOS*, *UBAC2-AS1*, *UBR5-DT*, ***ENSG00000236529***, ***ENSG00000261211***, ***ENSG00000268204***, ***ENSG00000272275***, ***AQP5-AS1***, ***ENSG00000272763***, ***ENSG00000261068***, *ENSG00000261135*, *ENSG00000261061*, *ENSG00000272872*, *ENSG00000224272*, *ENSG00000275216*, *ENSG00000225335*, *ENSG00000236924*, *ENSG00000250899*, *ENSG00000269155*, *ENSG00000245719*, *ENSG00000256234*, *ENSG00000259163*, *ENSG00000225489*, *ENSG00000260920*, *ENSG00000255114*, *ENSG00000270460*, *ENSG00000278238*, *ENSG00000267698*, *ENSG00000233461*, *ENSG00000270174*, *ENSG00000253174*, *ENSG00000269968*, *ENSG00000231295*, *ENSG00000232386*, *ENSG00000269399*, *ENSG00000226334*, *ENSG00000265415*, *ENSG00000234694*, *ENSG00000243224*, *ENSG00000267512*, *ENSG00000244184*, *ENSG00000261071*

**Table 2:** Downregulated lncRNAs in ovarian cancer tissue in at least three different transcriptomic studies from the “Diagnostic” analysis. For genes in bold, the position of the non-cancerous cell line OELE matches the fourth quartile when sorting the cell lines in ascending normalized counts.

Number of Studies	Gene Count	Gene Name
**15**	1	***MAGI2-AS3***
**13**	2	*ADAMTS9-AS2*, ***HAND2-AS1***
**12**	4	*CRNDE*, *GAS1RR*, *MIR22HG*, ***PGM5-AS1***
**11**	4	*ADAMTS9-AS1*, *ENSG00000267058*, *EPM2A-DT*, ***NR2F1-AS1***
**10**	4	***GATA6-AS1***, ***GIHCG***, *KLF3-AS1*, *WDFY3-AS2*
**9**	6	*ATP2B1-AS1*, *ERVK13-1*, *GLIDR*, ***HHIP-AS1***, *HYMAI*, ***SNHG8***
**8**	9	*C2orf27A*, ***CLN8-AS1***, *DPP10-AS1*, *ENSG00000272447*, *EPB41L4A-AS1*, *FGF14-AS2*, ***MIR100HG***, *RASSF8-AS1*, *SP2-AS1*
**7**	6	*ID2-AS1*, ***LINC00667***, ***LINC00842***, *LINC02941*, *LINC03013*, *ZFAS1*
**6**	12	***CKMT2-AS1***, *DUBR*, *EGOT*, *FGD5-AS1*, *GABPB1-AS1*, ***LINC00924***, *LINC01616*, *PAN3-AS1*, *SDCBP2-AS1*, ***SLC25A21-AS1***, ***SNHG18***, ***ZEB1-AS1***
**5**	13	*CERNA1*, *CPVL-AS2*, *ENSG00000204814*, ***FRMD6-AS2***, *LINC00342*, ***LINC00526***, *LINC00683*, *LINC02754*, ***MEF2C-AS1***, *NIFK-AS1*, *PAXBP1-AS1*, *PWRN1*, *VLDLR-AS1*
**4**	44	*ADD3-AS1*, *ANXA2R-AS1*, ***ARHGEF26-AS1***, *CRIM1-DT*, *DPH1-AS1*, *ENSG00000145075*, *ENSG00000204666*, ***ENSG00000230393***, *ENSG00000244953*, *ENSG00000245025*, *ENSG00000248115*, *ENSG00000253123*, *ENSG00000255495*, *ENSG00000258181*, *ENSG00000259976*, *ENSG00000261305*, *ENSG00000261671*, *ENSG00000270589*, *FGF13-AS1*, ***FLRT2-AS1***, *GASK1B-AS1*, *KLRK1-AS1*, *LETR1*, *LINC00324*, *LINC00926*, *LINC00997*, *LINC01018*, *LINC01391*, ***LINC02360***, *LNCRNA-IUR*, *MIR3936HG*, ***MRGPRF-AS1***, *NPEPPSP1*, ***NR2F2-AS1***, *NR4A1AS*, *OR2A1-AS1*, *PCAT19*, *PTPRD-AS1*, *RPH3AL-AS1*, *SNHG26*, *TMEM220-AS1*, *TPT1-AS1*, ***TRHDE-AS1***, ***WARS2-IT1***
**3**	138	*CAVIN2-AS1*, *CEROX1*, ***CNN3-DT***, *DHDDS-AS1*, *DNAJC27-AS1*, ***ELOA-AS1***, *ENSG00000226622*, *ENSG00000226862*, *ENSG00000227733*, *ENSG00000231609*, *ENSG00000232934*, *ENSG00000233178*, *ENSG00000233760*, *ENSG00000233894*, ***ENSG00000234394***, *ENSG00000234699*, ***ENSG00000235159***, ***ENSG00000235563***, *ENSG00000238018*, ***ENSG00000243004***, *ENSG00000246250*, ***ENSG00000249679***, *ENSG00000249849*, *ENSG00000250286*, *ENSG00000250326*, *ENSG00000254141*, *ENSG00000254855*, *ENSG00000256139*, *ENSG00000256973*, ***ENSG00000257894***, *ENSG00000259275*, *ENSG00000259359*, *ENSG00000259367*, *ENSG00000259969*, ***ENSG00000260269***, ***ENSG00000260277***, ***ENSG00000260563***, *ENSG00000260583*, *ENSG00000260645*, *ENSG00000260686*, *ENSG00000260693*, *ENSG00000260859*, *ENSG00000261167*, *ENSG00000261269*, *ENSG00000261292*, ***ENSG00000266677***, *ENSG00000267042*, *ENSG00000267082*, *ENSG00000267636*, ***ENSG00000267774***, *ENSG00000268047*, *ENSG00000268912*, *ENSG00000269068*, *ENSG00000269194*, *ENSG00000269210*, *ENSG00000270096*, *ENSG00000270140*, *ENSG00000270640*, *ENSG00000271730*, *ENSG00000271930*, *ENSG00000272158*, ***ENSG00000272159***, ***ENSG00000272823***, *ENSG00000272831*, *ENSG00000273650*, *ENSG00000274719*, *ENSG00000275120*, *ENSG00000277351*, *ENSG00000277954*, *ENTPD1-AS1*, *FAM167A-AS1*, *FAM182A*, *FAM53B-AS1*, *FAM66A*, *FAM85B*, *FOXO6-AS1*, *GARS1-DT*, ***GCC2-AS1***, *HOXA-AS2*, ***HSD11B1-AS1***, *INE2*, *IRS4-AS1*, *JAZF1-AS1*, *LINC00602*, *LINC00844*, ***LINC00847***, *LINC00886*, *LINC00891*, ***LINC00921***, *LINC01229*, *LINC01402*, *LINC01474*, *LINC01560*, *LINC01619*, *LINC01625*, ***LINC01852***, *LINC02126*, *LINC02145*, ***LINC02202***, *LINC02308*, *LINC02345*, *LINC02613*, *LINC02691*, *LINC02731*, *LINC03007*, *LRRC8C-DT*, ***LRRK2-DT***, *MAFTRR*, *MAP3K4-AS1*, *MEG8*, *MIMT1*, *MIOS-DT*, *MIR223HG*, ***MIR497HG***, *MRPS30-DT*, *MSC-AS1*, ***NALT1***, ***PACERR***, ***PGM5P3-AS1***, ***PGM5P4-AS1***, *PRR34-AS1*, *PRSS23-AS1*, *PSMG3-AS1*, *RASGRF2-AS1*, *RBPMS-AS1*, *RORA-AS1*, *SAP30-DT*, *SEMA6A-AS1*, *SMC5-DT*, *SNHG5*, *TICAM2-AS1*, ***TMC3-AS1***, *TSPEAR-AS1*, *TTC3-AS1*, *XPC-AS1*, ***ZEB2-AS1***, *ZNF300P1*, *ZNF594-DT*

**Table 3:** LncRNA genes positively correlated with longer (left) or shorter (right) OS periods in EOC patients identified in the studies from the “Prognosis” analysis. LncRNAs in bold are present in two different studies.

LncRNAs Correlating with Longer OS	LncRNAs Correlating with Shorter OS
***RNF157-AS1***, ***AQP5-AS1***, *AKAP1-DT*, *ANKRD44-AS1*, *ARHGAP28-AS1*, *BMPR1B-DT*, *CD2AP-DT*, *CEACAM16-AS1*, *DCTN6-DT*, *DNAJC9-AS1*, *DNM1P35*, *DTD1-AS1*, *EBLN3P*, *FAM27C*, *FOXP4-AS1*, *FZD4-DT*, *GPRC5D-AS1*, *HAGLROS*, *HCG14*, *HCP5*, *HLA-L*, *IFNG-AS1*, *IL6R-AS1*, *JARID2-DT*, *LINC00467*, *LINC00488*, *LINC00592*, *LINC00664*, *LINC01431*, *LINC01635*, *LINC01695*, *LINC01829*, *LINC01970*, *LINC02006*, *LINC02073*, *LINC02091*, *LINC02167*, *LINC02321*, *LINC02346*, *LINC02629*, *LINC02754*, *LINC02777*, *LUNAR1*, *MIR762HG*, *MYCNOS*, *MYL12-AS1*, *NUTM2A-AS1*, *PCAT18*, *POLH-AS1*, *RAB11B-AS1*, *RFX5-AS1*, *RGMB-AS1*, *RMST*, *RNASEH1-AS1*, *SCIRT*, *SNHG10*, *SRD5A3-AS1*, *TRBV11-2*, *TWSG1-DT*, *USP30-AS1*, *ENSG00000228863*, *ENSG00000259834*, *ENSG00000235021*, *ENSG00000273221*, *ENSG00000232739*, *ENSG00000270087*, *ENSG00000255135*, *ENSG00000256101*, *ENSG00000256948*, *SLC38A4-AS1*, *ENSG00000275769*, *ENSG00000276727*, *ENSG00000277863*, *ENSG00000258521*, *ENSG00000278022*, *ENSG00000259772*, *ENSG00000276408*, *ENSG00000263063*, *ENSG00000263427*, *ENSG00000265840*, *ENSG00000274213*, *ENSG00000277597*, *ENSG00000266149*, *ENSG00000267627*, *ENSG00000273368*, *ENSG00000268555*, *ENSG00000268650*, *ENSG00000230432*, *ENSG00000235319*, *ENSG00000273063*, *ENSG00000276517*, *ENSG00000281920*, *ENSG00000277901*, *ENSG00000273210*, *ENSG00000272858*, *ENSG00000250039*, *ENSG00000272626*, *ENSG00000272986*, *ENSG00000272203*, *ENSG00000272417*, *ENSG00000261071*, *ENSG00000272209*, *ENSG00000228506*, *ENSG00000261189*, *ENSG00000272009*, *ENSG00000272243*, *ENSG00000272379*, *ENSG00000231794*, *ENSG00000214870*, *ENSG00000237773*, *ENSG00000260997*, *ENSG00000273391*, *ENSG00000253982*, *PPP1R3B-DT*, *ENSG00000253369*, *ENSG00000254812*, *ENSG00000260484*, *ENSG00000253400*, *ENSG00000232412*, *ENSG00000290574*, *ENSG00000290689*, *ENSG00000290796*	***CRNDE***, ***ZFAS1***, *C2orf27A*, *DUXAP8*, *FLG-AS1*, *GUSBP11*, *LINC00484*, *LINC01127*, *LINC02881*, *MAGI2-AS3*, *MIR600HG*, *MIR924HG*, *NRSN2-AS1*, *PAXBP1-AS1*, *PCAT4*, *PIK3R5-DT*, *RP9P*, *ENSG00000260917*, *ENSG00000257545*, *ENSG00000259341*, *ENSG00000261069*, *ENSG00000271725*, *ENSG00000270020*, *ENSG00000275438*, *ENSG00000266709*, *LINC02958*, *ENSG00000213963*, *ENSG00000229839*, *ENSG00000270696*, *ENSG00000240057*, *ENSG00000230074*, *ENSG00000290659*

**Table 4:** LncRNA genes positively correlated with longer (left) or shorter (right) DFS periods in EOC patients identified in the studies from the “Prognosis” analysis. LncRNAs in bold are present in two different studies.

LncRNAs Correlating with Longer DFS	LncRNAs Correlating with Shorter DFS
*ADCY10P1*, *ANO1-AS1*, *ATXN2-AS*, *B4GALT1-AS1*, *C1orf21-DT*, *C6orf223*, *CRTC3-AS1*, *CT62*, *CXXC5-AS1*, *DIAPH2-AS1*, *DLGAP1-AS1*, *DNM1P35*, *EIF1B-AS1*, *EML2-AS1*, *FAM111A-DT*, *FAM27C*, *FOXP4-AS1*, *FZD4-DT*, *HCG15*, *IFNG-AS1*, *JARID2-DT*, *LINC00221*, *LINC00243*, *LINC00582*, *LINC00592*, *LINC00927*, *LINC01003*, *LINC01144*, *LINC01825*, *LINC02298*, *LINC02397*, *LINC02541*, *LINC02601*, *LINC02875*, *LL22NC03-63E9.3*, *LUNAR1*, *MAN2A1-DT*, *MIAT*, *MIR3142HG*, *NCKAP5-AS2*, *NDUFV2-AS1*, *NIFK-AS1*, *NR2F2-AS1*, *NSMCE1-DT*, *OLMALINC*, *PKP4-AS1*, *POLG-DT*, *PP2672*, *PPP1R21-DT*, *PPP1R26-AS1*, *RNF157-AS1*, *RORA-AS1*, *RPH3AL-AS1*, *SBF2-AS1*, *SLC25A5-AS1*, *SUGCT-AS1*, *TAX1BP1-AS1*, *THOC7-AS1*, *TRBV11-2*, *XIAP-AS1*, *ZSCAN5A-AS1*, *ENSG00000224152*, *ENSG00000226334*, *ENSG00000226889*, *ENSG00000228021*, *ENSG00000228084*, *ENSG00000228863*, *ENSG00000229311*, *ENSG00000230912*, *ENSG00000231081*, *ENSG00000231098*, *ENSG00000231519*, *ENSG00000231794*, *ENSG00000232533*, *ENSG00000233230*, *ENSG00000233242*, *ENSG00000234134*, *ENSG00000235586*, *ENSG00000236140*, *ENSG00000254290*, *ENSG00000254343*, *ENSG00000255440*, *ENSG00000257042*, *ENSG00000257327*, *AQP5-AS1*, *ENSG00000258534*, *ENSG00000258572*, *ENSG00000258904*, *ENSG00000259802*, *ENSG00000259834*, *ENSG00000260038*, *ENSG00000260352*, *ENSG00000260369*, *ENSG00000261071*, *ENSG00000261204*, *ENSG00000261320*, *ENSG00000261655*, *ENSG00000262115*, *ENSG00000263063*, *ENSG00000266830*, *ENSG00000267255*, *ENSG00000267666*, *ENSG00000269951*, *ENSG00000270115*, *ENSG00000270265*, *ENSG00000271367*, *ENSG00000272172*, *ENSG00000272209*, *ENSG00000272456*, *ENSG00000272672*, *ENSG00000272831*, *ENSG00000273004*, *ENSG00000273104*, *ENSG00000273162*, *ENSG00000273210*, *ENSG00000273456*, *ENSG00000273989*, *ENSG00000275155*, *ENSG00000275202*, *ENSG00000275297*, *ENSG00000275484*, *ENSG00000276408*, *ENSG00000277901*, *ENSG00000278022*, *ENSG00000291006*, *ENSG00000291136*	*GUSBP11*, ***MALAT1***, *AQP4-AS1*, *DSG2-AS1*, *HLA-F-AS1*, *HOTAIRM1*, *LINC00452*, *LINC00472*, *LINC00565*, *LINC01140*, *LINC02328*, *LINC02432*, *LINC-PINT*, *LNCOC1*, *MIR924HG*, *PHACTR2-AS1*, *RRP7BP*, *TMEM161B-AS1*, *ZNF295-AS1*, *ZNF503-AS2*, *ENSG00000233834*, *ENSG00000243004*, *ENSG00000248268*, *ENSG00000249476*, *ENSG00000258603*, *ENSG00000259065*, *ENSG00000260412*, *ENSG00000261292*, *ENSG00000265975*, *ENSG00000266283*, *ENSG00000270074*, *ENSG00000272632*, *ENSG00000278668*, *ENSG00000280604*

**Table 5:** Upregulated lncRNAs in metastasis in two or three patient transcriptomic studies, or in one patient transcriptomic study and also in metastatic versus primary tumor cell line groups from CCLE. Genes in bold coincide with significant differentially expressed (DE) lncRNAs between the primary and metastatic cell line groups using DESeq2, and underlined genes coincide with those that were significant in the receiver operating curve analyses.

Number of Studies	Number of Comparisons	Gene Count	Gene Name
3	4	2	*LINC02544*, *LINC01235*
2	3	2	*HECW2-AS1*, *MIR31HG*
2	2	20	***ENSG00000282057***, *UNC5C-AS1*, *ENSG00000261327*, *ENSG00000259807*, *MEG9*, *LINC01561*, *SNHG18*, *HOTAIRM1*, *LINC00922*, *LINC01619*, *FAM225A*, *HOXA-AS3*, *LINC00968*, *ENSG00000271811*, *ENSG00000272755*, *ENSG00000233682*, *ENSG00000248540*, *ENSG00000259663*, *LINC02593*, *HOXA-AS2*
1 + cell line	1	6	*TENM3-AS1*, *LINC01678*, *ENSG00000250410*, *ENSG00000261604*, ***ENSG0000022749***, ***LNCOG***

**Table 6:** Downregulated lncRNAs in one patient transcriptomic study and also in metastatic versus primary tumor cell line groups from CCLE. Genes in bold coincide with significant DE lncRNAs between the primary and metastatic cell line groups using DESeq2, and underlined genes coincide with those that were significant in the receiver operating curve analyses.

Number of Studies	Number of Comparisons	Gene Count	Gene Name
1 + cell line	1	8	*ENSG00000260604*, *ENSG00000267284*, *LINC01508*, *LINC01138*, *FAM160A1-DT*, *ENSG00000255118*, *ENSG00000249049*, ***ENSG00000225649***

**Table 7:** “Switch” lncRNAs in metastasis patient transcriptomic studies.

Status in Ascites	Gene Count	Gene Name
Up	13	*TP53TG1*, *ENSG00000253982*, *MIR210HG*, *LINC00667*, *ATP2B1-AS1*, *ENSG00000275210*, *LINC00847*, *ENSG00000259153*, *ENSG00000243655*, *FCGR1BP*, *LINC00888*, *ENSG00000291107*, *ENSG00000291230*
Down	13	*MEG3*, *PCBP1-AS1*, *ENSG00000223774*, *FLJ16779*, *ENSG00000263065*, *SNHG12*, *IGFBP7-AS1*, *IDI2-AS1*, *PSMA3-AS1*, *NUTM2B-AS1*, *SNHG3*, *MALAT1*, *SMG1P5*

**Table 8:** Differentially expressed lncRNAs in high-grade serous ovarian cancer patients identified in two or three (*) studies.

HGSOC	
Upregulated lncRNA Genes	Downregulated lncRNA Genes
*WT1-AS* *, *PART1*, *ENSG00000255135*	*FAM155A-IT1*, *DLX6-AS1*

**Table 9:** Differentially expressed lncRNAs in clear cell serous ovarian cancer patients identified in two, three (*) or four (in bold) studies.

CCOC	
Upregulated lncRNA Genes	Downregulated lncRNA Genes
**SNHG12**, **LINC00472**, *C8orf31* *, *RBPMS-AS1*, *CPNE8-AS1*, *FAM155A-IT1*, *LINC01137*, *LINC02765*, *LINC01637*, *PPP1R3B-DT*, *PCCA-DT*, *LINC00598*, *LINC02435*, *ENSG00000271992*, *ENSG00000259052*, *ENSG00000218416*, *ENSG00000253307*, *ENSG00000223786*, *ENSG00000224583*, *ENSG00000264596*, *ENSG00000274718*, *ENSG00000187185*, *ENSG00000253666*, *ENSG00000257681*, *ENSG00000257831*, *ENSG00000236283*, *ENSG00000260317*, *ENSG00000265625*, *ENSG00000286546*, *ENSG00000249125*	*PART1*, *ZNF667-AS1*, *WT1-AS*, *HCP5*, *LINC01139*, *TARID*, *PAXIP1-DT PRKCQ-AS1*, *EMX2OS*, *ENSG00000227733*, *ENSG00000235560*, *ENSG00000255135*

## Data Availability

Data is contained within the article or Supplementary Material.

## Supplementary Materials

The supporting information can be downloaded at: https://www.mdpi.com/article/10.3390/ijms241310798/s1. Complete results of the meta-analysis and references of previous studies [120,121,122,123,124,125,126,127,128,129,130,131,132,133,134,135,136,137,138,139,140,141,142,143,144,145,146,147,148,149,150,151,152,153,154,155,156,157,158,159,160,161,162,163,164,165,166,167,168,169,170,171,172,173,174,175,176,177,178,179,180,181,182,183,184,185,186,187,188,189,190,191,192,193,194,195,196,197,198,199,200,201,202,203,204,205,206,207,208,209,210,211,212,213,214,215,216,217,218,219,220,221,222,223,224,225,226,227,228,229,230,231,232,233,234,235,236,237,238,239,240,241,242,243,244,245,246,247,248,249,250,251,252,253,254,255,256,257,258,259,260,261,262,263,264,265,266,267,268,269,270,271,272,273,274,275,276,277,278,279,280,281,282,283,284,285,286,287,288,289,290,291,292,293,294,295,296,297,298,299,300,301,302,303,304,305,306,307,308,309,310,311].
