# Supplementary material for: Identification of lncRNAs Deregulated in Epithelial Ovarian Cancer Based on a Gene Expression Profiling Meta-Analysis
Source: Int J Mol Sci. 2023 Jun 28;24(13):10798. doi: 10.3390/ijms241310798 (PMC10341812; doi:10.3390/ijms241310798)

Supplementary Figure S1.- Expression in other tissues of the two potential tumor suppressors identified in the meta-analysis . Information is provided from GEPIA, which compares TCGA (tumor) information and GTEx (normal). The upper bar graph corresponds to *NR2F2-AS1* and the lower to *RPH3AL-AS1*. TPM, transcripts per million.

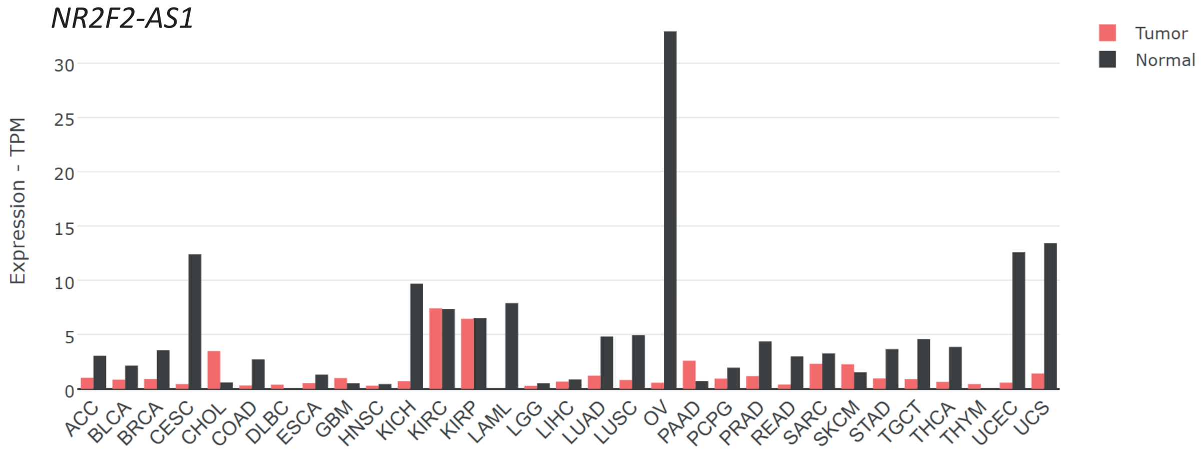

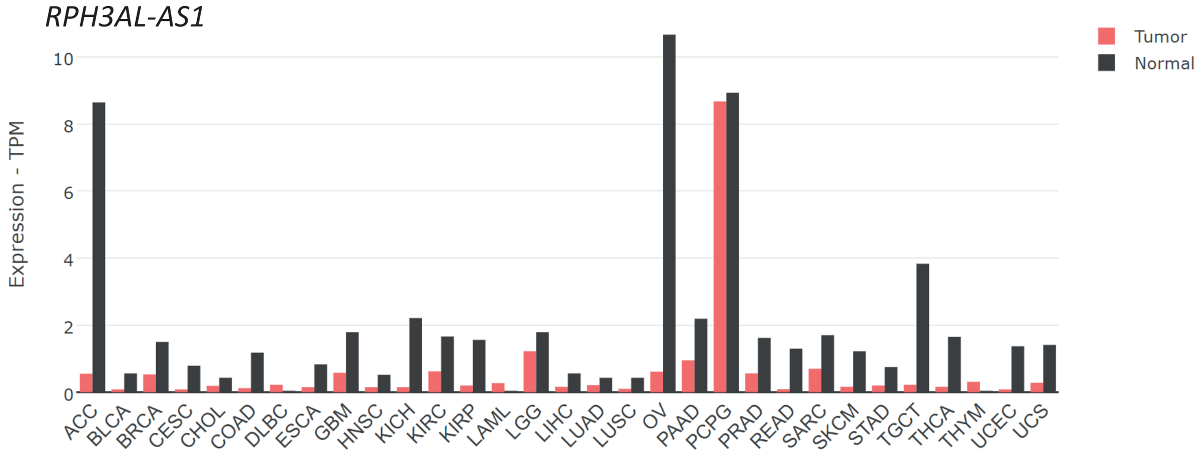

Supplement: Supplementary file 1 [file ijms-24-10798-s001.zip › Supplementary Figure S1.pdf]
